# Year-to-year Variability in Arctic Minimum Sea Ice Extent and its Preconditions in Observations and the CESM Large Ensemble Simulations

**DOI:** 10.1038/s41598-018-27149-y

**Published:** 2018-06-13

**Authors:** Wenchang Yang, Gudrun Magnusdottir

**Affiliations:** Department of Earth System Science, University of California, Irvine, USA

## Abstract

Arctic sea ice extent (SIE) achieves its minimum in September each year and this value has been observed to decline steeply over the satellite era of the past three decades. Yet large year-to-year fluctuations are also present in the September SIE and the mechanisms for this variability are still not clear. Here we address this issue by examining the preconditions in meteorological fields in the previous spring and summer from observations and a large ensemble of historical climate model simulations. The focus of this study is on the impact of anomalous moisture transport into the Arctic and the associated surface energy fluxes on the September SIE. We find that the below-normal September SIE is associated with enhanced moisture transport into the Arctic in spring, which induces downward thermal radiation at the surface. However, in summer, the anomalous moisture transport over the Arctic is divergent due to an anticyclonic atmospheric flow pattern and the ice albedo feedback plays a leading role in sea ice loss.

## Introduction

**Figure 1:**
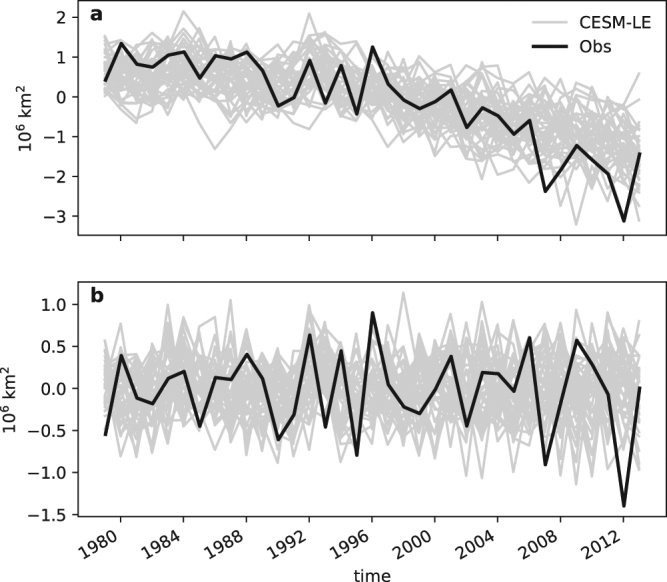
Northern Hemisphere September sea-ice extent anomalies from observation (black line) as well as 40 members of CESM-LE (gray lines). The sea-ice extent is defined as the total area of grid cells with sea ice concentration greater than 15%. The anomaly is defined as the deviation from the 1981–2010 climatology. (**a**) The raw time series. (**b**) Same as (**a**) except being highpass-filtered with a cutoff period of 9 years.

Arctic Amplification (AA) is one of the most dramatic climate trends taking place over recent decades, as the mean surface temperature (especially in winter) has warmed twice as fast as the rest of the Earth^1,2^. At the same time, the Arctic area covered by sea ice, or the Arctic sea ice extent (SIE), has also declined rapidly^3,4^, especially in September when the SIE reaches its annual minimum (Fig. 1). Besides AA and the rapid Arctic SIE decline, there is also substantial year-to-year variability of SIE superimposed on the long-term trend^5,6^. Yet, this interannual variability has received substantially less attention than the long-term trend.

Potential drivers of the SIE interannual variability are either from the ocean^7,8^ or the atmospheric circulation^8,9^ or both. The atmospheric circulation can impact sea ice both dynamically through the change in surface winds and thermodynamically through the associated transport of heat and moisture into the Arctic^10–14^. Therefore, it has been hypothesized that anomalously high moisture transport into the Arctic in spring, which is usually accompanied by both intense surface winds and a burst of high moisture content and warming within the Arctic atmospheric column, will lead to a decreased Arctic SIE in September^5^. Indeed, recent observational studies show that in years when there is low sea ice concentration (SIC) in summer and autumn, there is increased moisture transport into the Arctic in the preceding spring^5,6^.

It is still unclear if the relationship between moisture transport into the Arctic and SIC revealed in observations can be reproduced in Global Climate Model (GCM) simulations. The observations also do not allow for analysis of the uncertainty in the relationship between moisture transport and SIE due to internal variability since the observed climate is just one representation in a range of possible climate states. We will address these issues in the current study. Specifically, we examine the correlation between the Northern Hemisphere September SIE and moisture transport into the Arctic in the preceding spring and summer seasons by analyzing model output from 40 ensemble members of the Community Earth System Model Large Ensemble (CESM-LE) Project^15^. We compare the results from CESM-LE to observations and also examine the uncertainty of the relationship by examining the spread of the results from the 40 ensemble members.

## Northern Hemisphere September SIE

Figure 1a shows the Northern Hemisphere September SIE anomalies from observations and 40 ensemble members of CESM-LE. A declining trend since the late 1970s is apparent in both observations and CESM-LE, with a decrease of 2–3 million square kilometers during the period of 1979–2013. In addition to the long-term trend we can see large year-to-year fluctuations, and the amplitude in observations is comparable to that in CESM-LE. To focus on this interannual variability, we apply a high-pass filter to all the raw time series of September SIE with a cutoff period of 9 years. The resulting time series, named SIE09hp henceforth (“09” and “hp” denote September and high-pass, respectively), is shown in Fig. 1b, in black for observations and in grey for the ensemble members. The CESM-LE time series also show a ~2 million square kilometer range of fluctuations, similar in magnitude to the multi-decade declining trend. Comparing results from observations to those from CESM-LE, it is clear that the observed SIE is in general within the range of internal variability as indicated by the spread of the 40 ensemble members from CESM-LE. Therefore, simulations from CESM-LE reproduce the key characteristics of the Northern Hemisphere SIE interannual variability.

## Spring and Summer Moisture Transport and Circulation

**Figure 2:**
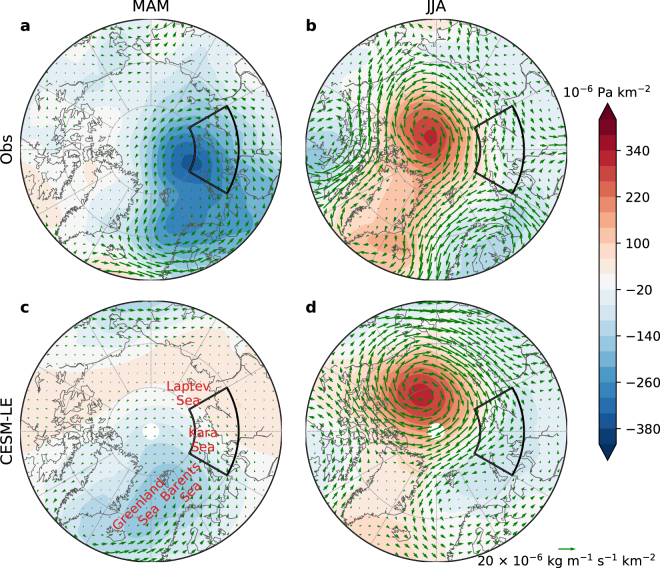
Seasonal-mean sea-level pressure (shadings) and moisture transport (vertically integrated moisture flux, vectors) associated with negative anomalies of high-pass-filtered Northern Hemisphere sea-ice extent in September (SIE09hp) as estimated from linear regression. (**a**,**b**) Are from observations, where the sea-level pressure and moisture transport are from ERA-Interim reanalysis. (**c**,**d**) Are ensemble-mean regressions from CESM-LE. Left (right) column shows results from season MAM (JJA). The black longitude-latitude boxes outline the region of 60E–120E and 70N–80N. Maps were generated using the “matplotlib basemap toolkit” Python package (https://matplotlib.org/basemap/), version 1.1.0.

To reveal the spatial patterns of atmospheric fields associated with low September SIE on the interannual time scale, we conduct a linear regression analysis of these fields on the negative of the time series SIE09hp, or −SIE09hp. The regressed sea-level pressure and moisture transport are shown in Fig. 2, showing the results both in spring (March–May, MAM) and summer (June–August, JJA), and both from ERA-Interim reanalysis (henceforth, reanalysis) and CESM-LE. In spring, a low pressure pattern is centered around the Barents-Kara Seas in observations (Fig. 2a). Consistent with the low pressure pattern, moisture is transported into the Arctic through the Kara-Laptev Sea pathway and returned southward through the Greenland Sea longitudes. The low pressure pattern is also seen in CESM-LE (Fig. 2c), but it is located to the west of the observed one, between the Greenland and Barents Seas. Due to the westward shift of the low pressure center in CESM-LE, the moisture transport into the Arctic is now through the Barents and Kara Seas. A weak moisture transport back southward along the the Greenland Sea is also seen in CESM-LE, although the magnitude is smaller than in observations.

In summer, there is an anomalous high over the high Arctic both in reanalysis (Fig. 2b) and CESM-LE (Fig. 2d). Consequently, a clockwise moisture transport is seen around the anomalous high. As a result, the zonal direction of the moisture transport over the region of 60E–120E and 70N–80N (the black longitude-latitude box in Fig. 2) is opposite to that in the spring season.

The relationships between September Arctic SIE and the atmospheric circulation in spring and summer revealed in observations can be reproduced not only in the ensemble mean of different simulations from a single model (CESM-LE) but also in those of 30 different models from the Coupled Model Intercomparison Project Phase 5 (CMIP5) (Figs S1 and S2). For example, the spring low pressure pattern centered around the Barents-Kara Seas and the summer high pressure pattern over the Arctic are both reproduced well in the CMIP5 multi-model ensemble means (Fig. S2). Therefore, the general agreement between observations and model simulations regarding the relationship between spring and summer sea-level pressure and the September SIE is not restricted to one single model (CESM) but is robust for a large number of different climate models.

The regressed patterns shown above are not sensitive to which observational data sets are used (Fig. S3), and even not sensitive to which period is used for regression for CESM-LE (Fig. S4c,d). However, in ERA-20C reanalysis, the regressed patterns are significantly different before and after 1980 (Fig. S4a,b). This is primarily due to great uncertainty in the sea ice concentration estimates in the pre-satellite era. By looking at the regressed patterns in each individual month, we find that the spring SLP patterns are dominated by March and April both in reanalysis (Fig. S5) and CESM-LE (Fig. S6) In summer, observed moisture transport is much stronger in August than June but the CESM-LE values have a similar magnitude in all the three months. Finally, regressed patterns from sea-ice fields are generally consistent with the circulation patterns shown in Fig. 2 (see Fig. S7).

## Correlation with Moisture Transport

Next, we calculate the correlation coefficient between the moisture transport in each individual month and −SIE09hp to test the hypothesis that anomalously high spring moisture transport into the Arctic will lead to a low Arctic sea ice extent in September. Here, we define the index of moisture transport as the zonal component of vertically integrated moisture flux averaged over the region of 60E–120E and 70N–80N (the black longitude-latitude box in Fig. 2). The choice of this index as well as the regional box has multiple reasons. First, the zonal moisture transport is part of the cyclonic (spring) and anticyclonic (summer) patterns of moisture transport over the Arctic and thus more or less representative of the overall moisture transport. Second, it has opposite signs between spring and summer both in reanalysis and CESM-LE, which reflects the opposite patterns of moisture transport between the two seasons. Third, the index is highly correlated to the moisture transport through the Laptev Sea area, where the September sea ice concentration typically shows the strongest anomalies^5^. The fact that this choice leads to more consistent behavior with respect to surface fluxes as will be seen later further strengthens this point to some degree.

**Figure 3:**
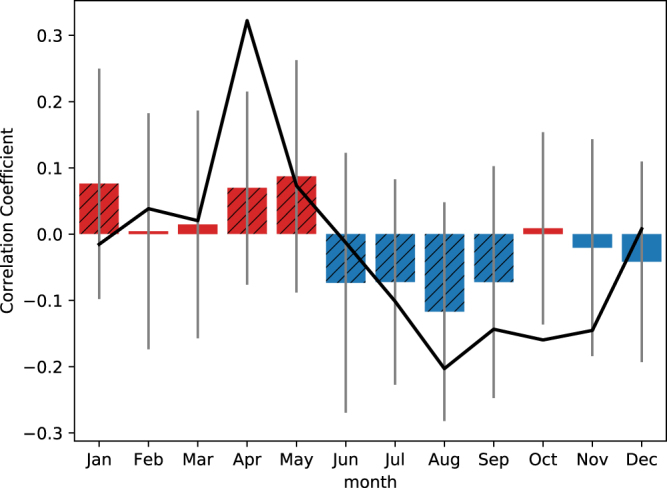
Correlation between the zonal component of vertically integrated moisture flux averaged over the region of 60E–120E and 70N–80N (the black longitude-latitude box in the previous figure) and −SIE09hp in observation (black line) and CESM-LE (bars and gray lines) for each month. The bars represent ensemble mean, while the vertical gray lines show the spread of one standard deviation from the ensemble members. Hatches denote ensemble-mean values significantly different from zero at 0.05 level by the two-sided Student’s t-test.

Figure 3 shows the correlation between the index and −SIE09hp in reanalysis (black line) and CESM-LE (bars and gray lines) as a function of calendar month. The leading feature from the observations is the contrast between spring and summer: while the moisture transport index is positively correlated with −SIE09hp, the correlation coefficient becomes negative in summer. The same feature can also be seen in the CESM-LE ensemble mean with positive and negative correlation coefficients in spring and summer, respectively, although the peak value month is April in reanalysis and May in CESM-LE. While the ensemble mean values are consistent with observations in spring and summer, there is indeed a large spread from the 40 ensemble members as indicated by the gray vertical lines in Fig. 3. For example, while the ensemble mean value has the maximum value in May, results from individual ensemble members can also be negative in the same month. Also, in August, when the ensemble mean is most negative, individual ensemble members can still have positive values, implying that moisture transport appears to have a modulating rather than a controlling role.

**Figure 4:**
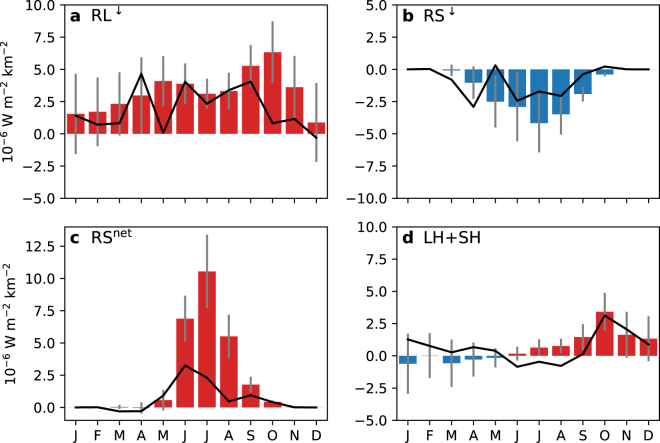
Regressed surface heat fluxes averaged over the Arctic (north of 70N) on the −SIE09hp for each month. (**a**–**d**) Are downward longwave radiation (RL^↓^), downward shortwave radiation (RS^↓^), net shortwave radiation (RS^net^), and latent plus sensible heat fluxes (LH + SH), respectively. The heat fluxes are defined positive downward in (**a**–**c**), but positive upward in (**d**).

We also examined the index of the zonal mean of meridional moisture transport at 70N (Fig. S8), which represents the moisture convergence into the Arctic, and has a similar correlation coefficient with −SIE09hp as the index used in our study, especially for CESM-LE. However, there are indeed some important differences: while the index used in the current study has the highest correlation coefficient with −SIE09hp in April (Fig. 3), the correlation peaks in March and May for the moisture convergence index in observation and is close to zero in April (Fig. S8). Our physically motivated box index shows the largest correlations with −SIE09hp in April, at the same time when the regressed surface heat fluxes averaged over the Arctic north of 70N are at a local maximum (Fig. 4).

## Surface Heat Fluxes

The dominant way in which the moisture transport can impact the Arctic SIE is by inducing anomalous vertical heat flux at the surface^5^. It is therefore crucial to examine the regressed surface heat flux on the Arctic SIE. Figure 4 shows different components of the surface heat flux regressed onto the −SIE09hp for the Arctic region north of 70N (The spatial patterns in observations and CESM-LE are shown in Figs S9 and S10, respectively). The downward longwave radiation (Fig. 4a) is in general positive both in observations and CESM-LE, including in the spring season. There is an opposite effect on the shortwave radiation^5^, indicated by the negative regressed values in spring in Fig. 4b. It is consistent with the hypothesis that spring moisture transport into the Arctic can induce anomalous downward longwave radiation at the surface that initiates the sea ice melt.

In the summer season, sea ice albedo feedback is a dominant mechanism, since the regressed downward shortwave radiation has negative values but the net shortwave radiation has large positive values. This can only be achieved by greatly reduced shortwave radiation reflected back upward at the surface, which is realized through the sea ice albedo feedback. This is consistent with the argument in previous studies^5^ that moisture transport initiates the sea ice melt in spring, and sea ice albedo feedback amplifies the melt in summer. The regressed latent plus sensible heat fluxes are generally weak in spring and summer, but become strong in September and peak in October. This is a result of the delayed response in the atmosphere to sea-ice loss as the surface and overlying lower atmosphere are close to thermodynamic equilibrium in summer, but that is not the case in fall and the effect is greater in October than in September^16^.

## Conclusion

It has been hypothesized that spring moisture transport into the Arctic can initiate sea ice melt and lead to below-normal SIE in September, when the Arctic SIE reaches its minimum of the year. The moisture transport could therefore be an important driver that can help explain the year-to-year fluctuations of the Arctic SIE minimum. Here we test this hypothesis using CESM-LE consisting of 40 identically forced simulations during the period 1979–2013. We find that low September SIE is linked to enhanced spring moisture transport into the Arctic and an anomalous downward thermal radiation at the surface in both observations and CESM-LE, which is consistent with the proposed hypothesis. Furthermore, we also see an anomalous moisture transport out of the Arctic in summer, which is associated with the anticyclonic atmospheric flow pattern centered around the North Pole. In contrast to our findings for spring, the ice albedo feedback is likely the dominant process that reduces sea ice in summer. The correlation between moisture transport and September SIE has opposite signs in spring and summer both in observations and the CESM-LE ensemble mean, due to different moisture transport directions in the two seasons. However, the correlation shows large internal variability as revealed by the spread of the results for individual ensemble members of CESM-LE, implying that moisture transport plays a modulating rather than a controlling role and other processes like sea ice export variability also have to be considered.

## Methods

### Data

We use the European Centre for Medium-Range Weather Forecasts (ECMWF) Interim (ERA-Interim) reanalysis^17^ for monthly atmospheric circulation, vertically integrated moisture transport and surface heat fluxes. For the surface heat fluxes, we first calculate the daily means from the 12-hour forecasts and then calculate the monthly means. To compare the results with an earlier period, we also use the ERA-20C reanalysis^18^, which is ECMWF’s atmospheric reanalysis of the 20th century (1900–2010) that assimilates observations of surface pressure and surface marine winds only. For observed sea ice concentration, we use version 1 of Gridded Monthly Sea Ice Extent and Concentration^19^ as the primary data set, and version 2 of NOAA/NSIDC Climate Data Record of Passive Microwave Sea Ice Concentration^20,21^ for comparison. We also use the observational sea ice motion data set from version 3 of Polar Pathfinder Daily 25 km EASE-Grid Sea Ice Motion Vectors^22^. For model outputs, we analyze 40 ensemble members of the Community Earth System Model Large Ensemble (CESM-LE) Project^15^. Each ensemble member of CESM-LE is a fully-coupled simulation with slightly different atmospheric initial conditions that is integrated from 1920 to 2100. These simulations are forced by historical forcing during 1920–2005 and RCP8.5 radiative forcing (or a scenario of comparatively high greenhouse gas emissions) thereafter. Our analysis is mainly focused on the period 1979–2013 to compare with satellite observations of SIE. Therefore, the CESM-LE simulations analyzed in this study include historical simulations over the years 1979–2005 plus RCP8.5 projections over the year 2006–2013. For a comparison, we also analyze outputs from the Coupled Model Intercomparison Project Phase 5 (CMIP5)^23^, for which we use 30 different models and pick one single simulation for each model (Table S1).

### September SIE Index

Sea ice extent (SIE) is defined as the total area covered by grid points that have the sea ice concentration (SIC) greater than 15%. In this study, our focus is on the September SIE over the Northern Hemisphere (north of 30N). In order to isolate the year-to-year variability of September SIE from the long-term trend, we apply a second-order forward-backward Butterworth high-pass filter with a cutoff period of 9 years. The resulting SIE time series, SIE09hp (“09” and “hp” denote September and high-pass, respectively), is then used in the linear regression analysis to reveal the relationship between the September SIE and other meteorological fields on the interannual time scale. In order to relate other fields directly with the September sea ice loss, the actual time series of September SIE used in the regression analysis is its negative, or −SIE09hp.

### Moisture Transport and the Index

The term of moisture transport used in this study refers to the vertically integrated moisture flux and is a horizontal vector field. The moisture transport index used in the correlation analysis is defined as the zonal component of the moisture transport averaged over the region of 60E–120E and 70N–80N (the longitude-latitude box in Fig. 2), which is more correlated with surface heat fluxes (Fig. 4) than the moisture convergence into the Arctic (Fig. S8).

### Figures

All the figures in the paper as well as the supplementary information file are created using the “matplotlib” Python package^24^.

### Data availability

ERA-Interim and ERA-20C data sets can be downloaded at http://apps.ecmwf.int/datasets/. The observed sea ice data can be downloaded at http://nsidc.org/data/G10010, http://nsidc.org/data/G02202/versions/2 and http://nsidc.org/data/nsidc-0116/versions/3. The CESM-LE model data can be downloaded at http://www.cesm.ucar.edu/projects/community-projects/LENS/data-sets.html and the CMIP5 data can be downloaded at http://cmip-pcmdi.llnl.gov/cmip5/data_portal.html.

## Electronic supplementary material

Supplementary Information
