# Supplementary material for: Year-to-year Variability in Arctic Minimum Sea Ice Extent and its Preconditions in Observations and the CESM Large Ensemble Simulations
Source: Sci Rep. 2018 Jun 13;8:9070. doi: 10.1038/s41598-018-27149-y (PMC5998069; doi:10.1038/s41598-018-27149-y)
Supplement: Supplementary file 1 — Supplementary Information [file 41598_2018_27149_MOESM1_ESM.pdf]

Supplementary Information for "Year-to-year Variability in Arctic Minimum Sea Ice Extent and its Preconditions in Observations and the CESM Large Ensemble Simulations"

Wenchang Yang[1], Gudrun Magnusdottir[1]

[1]*Department of Earth System Science, University of California, Irvine*

Table S1: The CMIP5 models used in this study.

Model Name	Ensemble Member
ACCESS1-0	r1i1p1
ACCESS1-3	r1i1p1
BCC-CSM1-1	r1i1p1
BCC-CSM1-1-M	r1i1p1
CCSM4	r1i1p1
CESM1-CAM5	r1i1p1
CESM1-WACCM	r2i1p1
CMCC-CM	r1i1p1
CNRM-CM5	r1i1p1
CSIRO-Mk3-6-0	r1i1p1
CanESM2	r1i1p1
FGOALS-g2	r1i1p1
GFDL-CM3	r1i1p1
GFDL-ESM2G	r1i1p1
GFDL-ESM2M	r1i1p1
GISS-E2-H	r1i1p1
GISS-E2-R	r1i1p1
HadGEM2-CC	r1i1p1
HadGEM2-ES	r1i1p1
INMCM4	r1i1p1
IPSL-CM5A-LR	r1i1p1
IPSL-CM5A-MR	r1i1p1
IPSL-CM5B-LR	r1i1p1
MIROC-ESM	r1i1p1
MIROC-ESM-CHEM	r1i1p1
MIROC5	r1i1p1
MPI-ESM-LR	r1i1p1
MPI-ESM-MR	r1i1p1
MRI-CGCM3	r1i1p1
NorESM1-M	r1i1p1

1. Walsh, J. E., Chapman, W. & Fetterer, F. Gridded monthly sea ice extent and concentration, 1850 onward, version 1. Boulder, Colorado USA. NSIDC: National Snow and Ice Data Center. (2015, updated 2016). https://doi.org/10.7265/N5833PZ5.

2. Meier, W. *et al.* NOAA/NSIDC climate data record of passive microwave sea ice concentration, version 2. Boulder, Colorado USA. NSIDC: National Snow and Ice Data Center. (2013, updated 2016). http://dx.doi.org/10.7265/N55M63M1.

3. Peng, G., Meier, W. N., Scott, D. J. & Savoie, M. H. A long-term and reproducible passive microwave sea ice concentration data record for climate studies and monitoring. *Earth System Science Data* **5**, 311–318 (2013). https://www.earth-syst-sci-data.net/5/311/2013/.

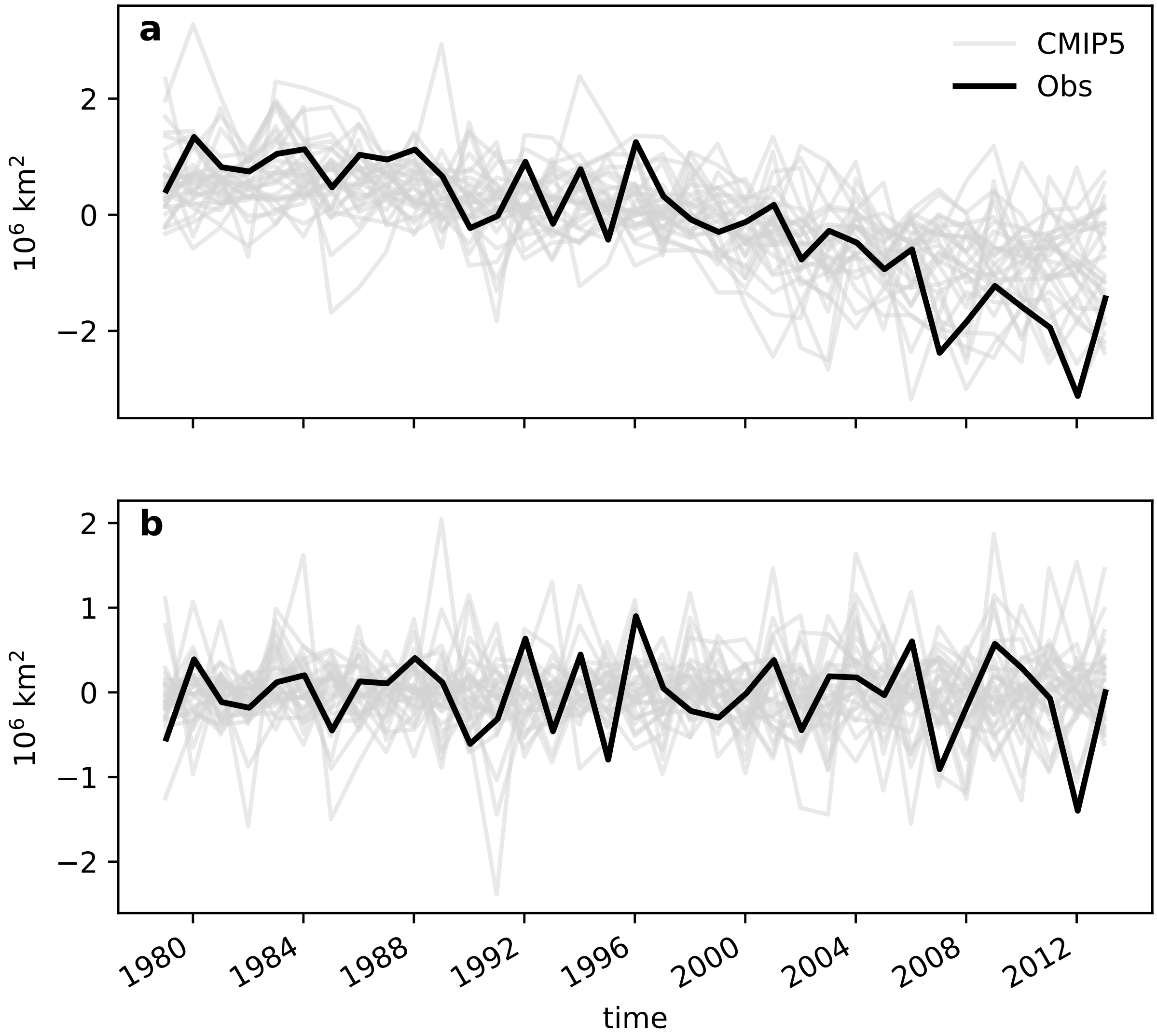

Figure S1: Northern Hemisphere September sea-ice extent anomalies from observation (black line) as well as 30 CMIP5 models (gray lines). The sea-ice extent is defined as the total area of grids with sea ice concentration greater than 15%. The anomaly is defined as the deviation from the 1981–2010 climatology. (a) The raw time series. (b) Same as (a) except being highpass-filtered with a cutoff period of 9 years.

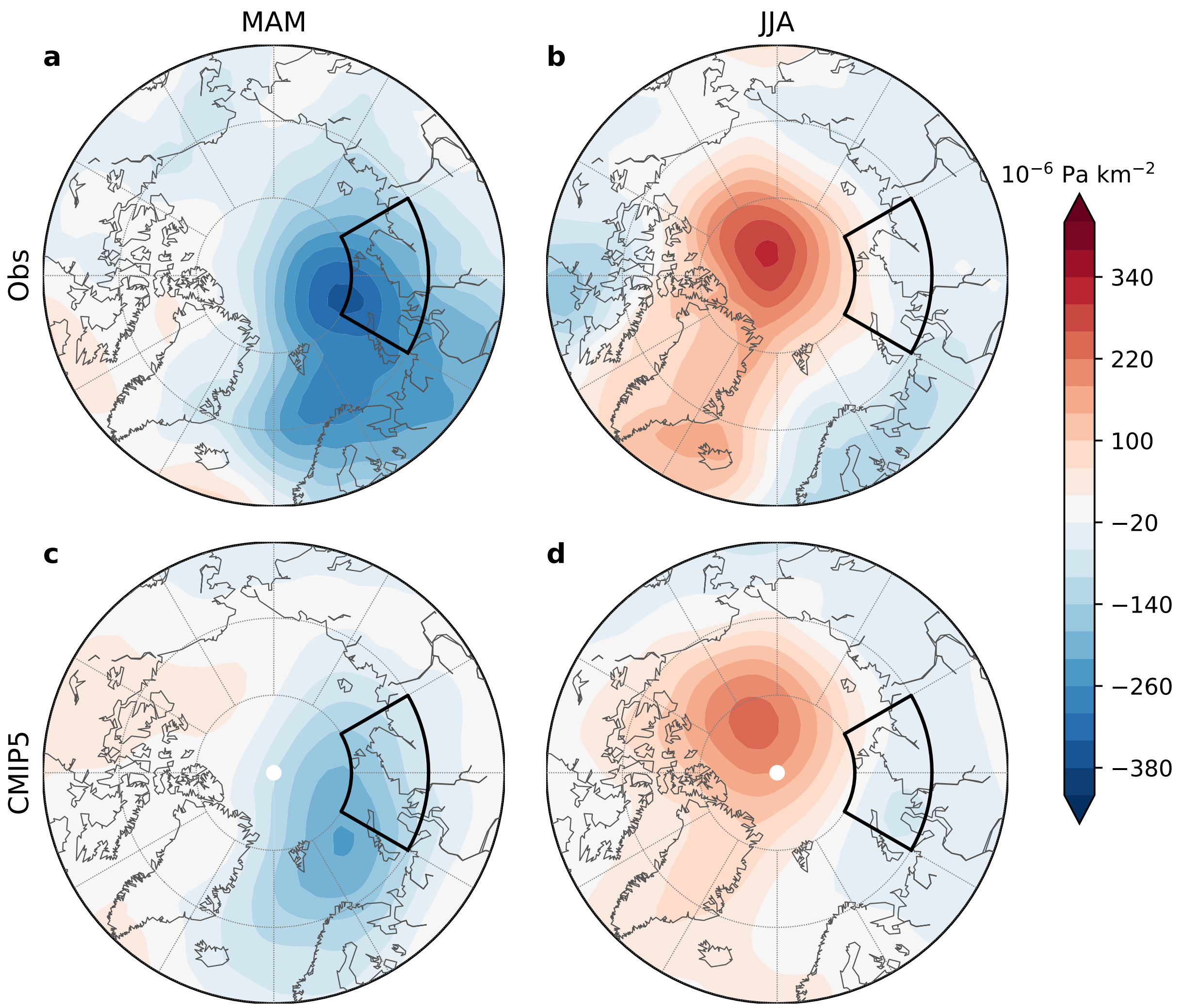

Figure S2: Seasonal-mean sea-level pressure associated with negative anomalies of high-pass-filtered Northern Hemisphere sea-ice extent in September (SIE09hp) as estimated from linear regression. (a) and (b) are from observations, where the sea-level pressure and moisture transport are from ERA-Interim reanalysis. (c) and (d) are from CMIP5 ensemble means. Left (right) column shows results from season MAM (JJA). The black longitude-latitude boxes outline the region of 60E-120E and 70N-80N. Maps were generated using the "matplotlib basemap toolkit" Python package (`https://matplotlib.org/basemap/`), version 1.1.0.

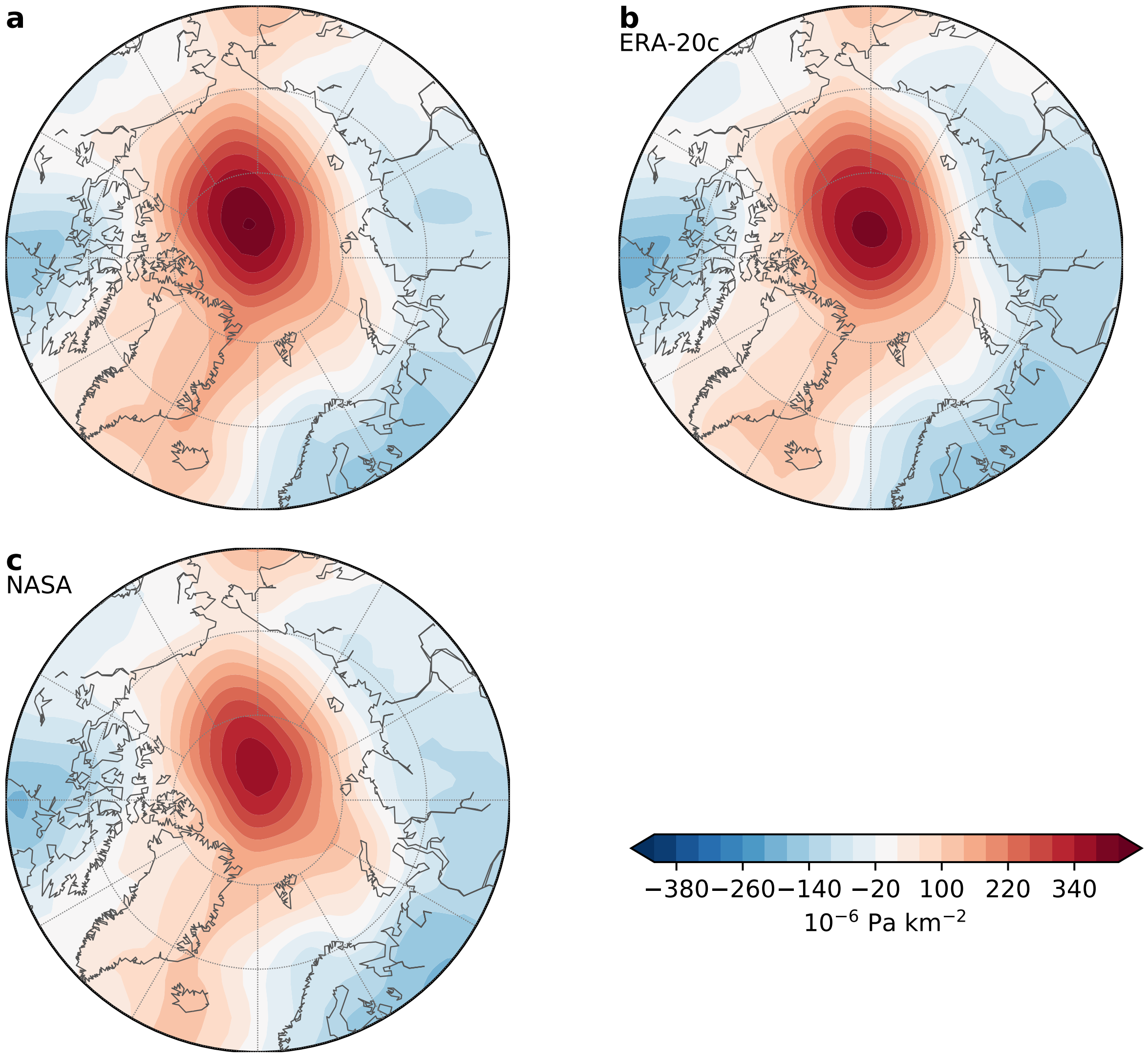

Figure S3: Regression of JJA sea level pressure (SLP) on −SIE09hp over 1981–2010. (a) The SLP is from the ERA-Interim reanalysis and the SIE is from Version 1 of Gridded Monthly Sea Ice Extent and Concentration [1]. (b) The SLP is from the ERA-20c reanalysis while the SIE has the same source as (a). (c) The SIE is from Version 2 of NOAA/NSIDC Climate Data Record of Passive Microwave Sea Ice Concentration [2,3] while the SLP has the same source as (a). Maps were generated using the "matplotlib basemap toolkit" Python package (`https://matplotlib.org/basemap/`), version 1.1.0.

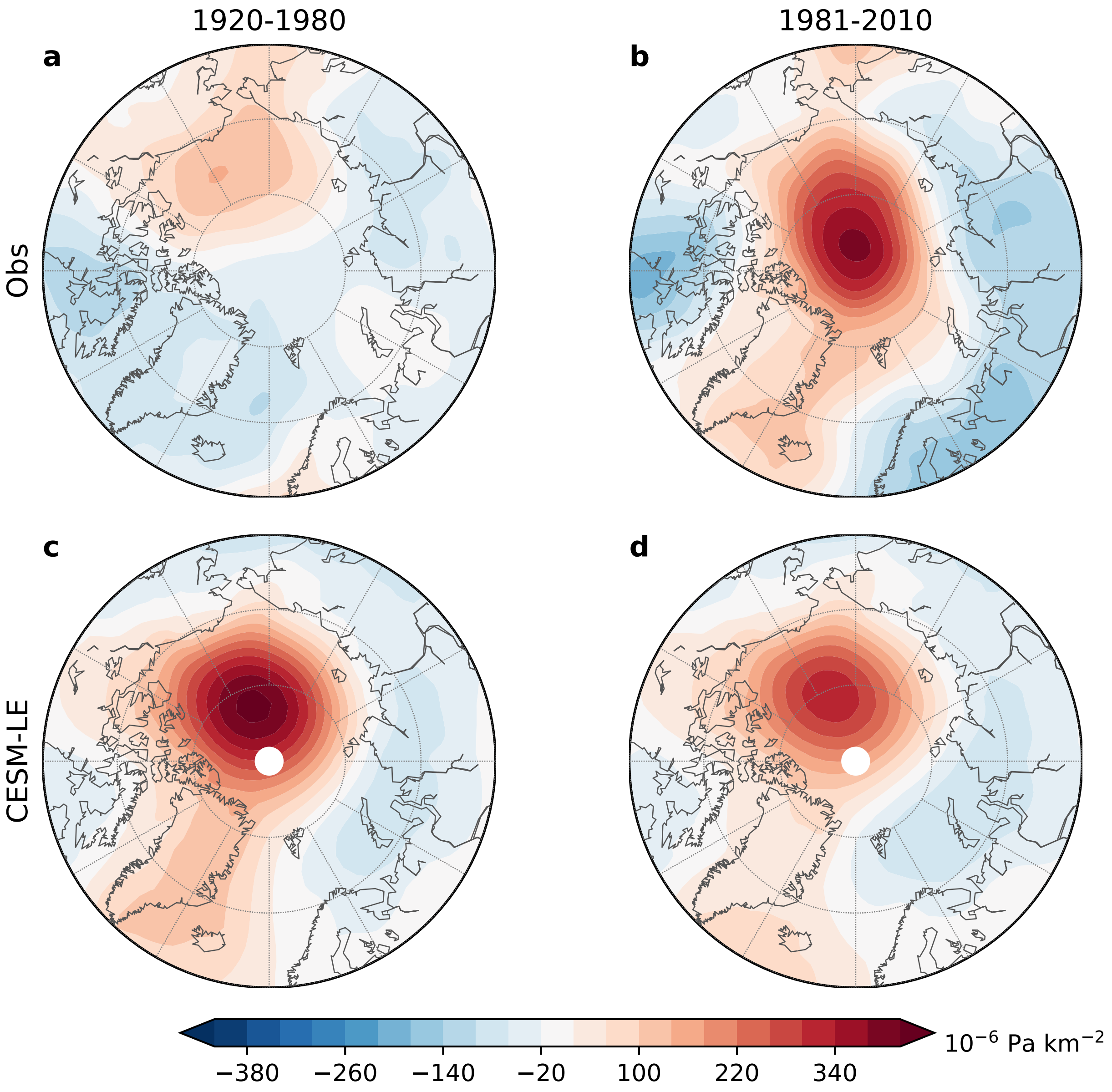

Figure S4: Regression of JJA SLP on −SIE09hp over the years of 1920–1980 (a and c) and 1981–2010 (b and d) for observations (a and b) and CESM-LE ensemble means (c and d). Maps were generated using the "matplotlib basemap toolkit" Python package (`https://matplotlib.org/basemap/`), version 1.1.0.

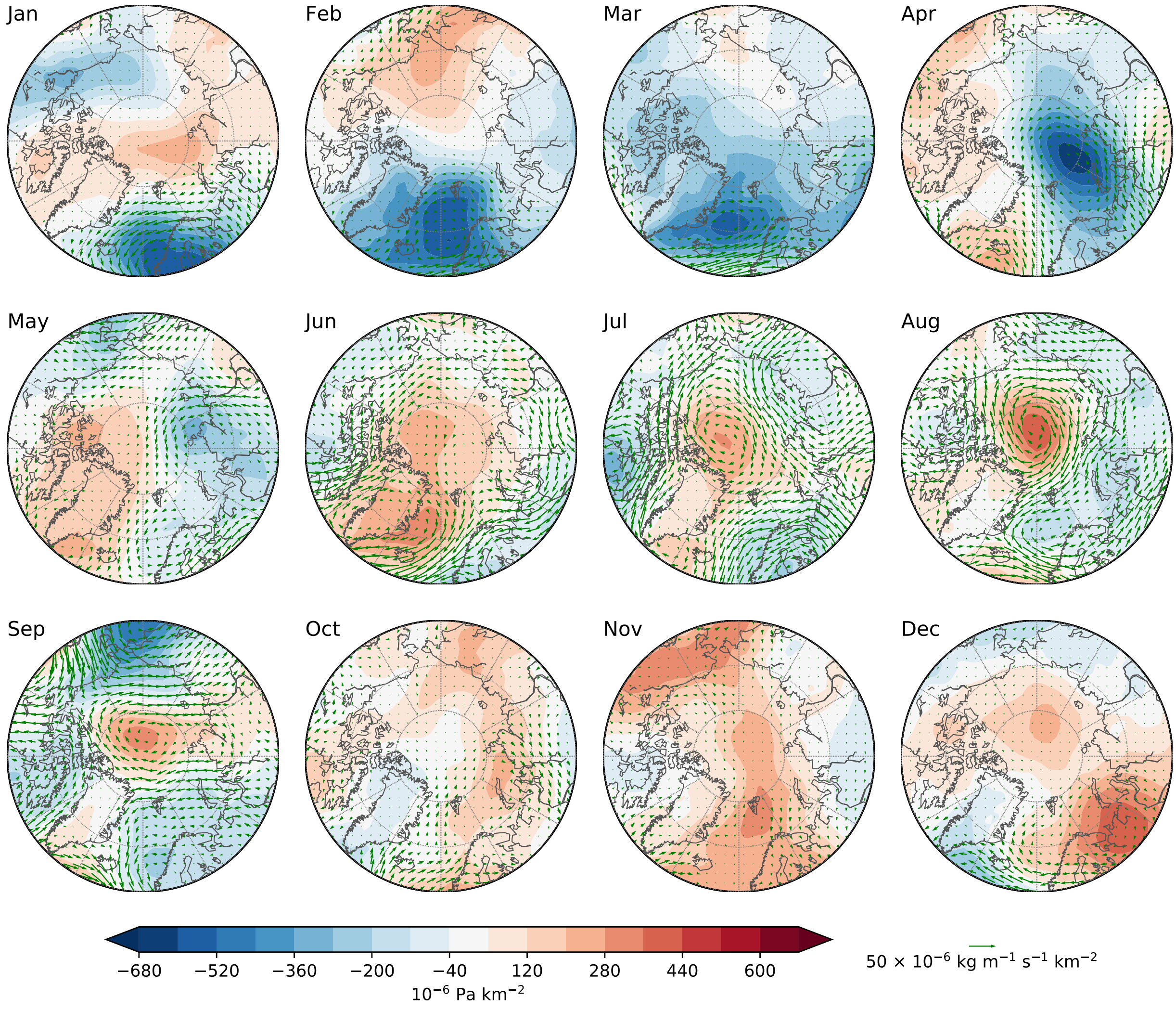

Figure S5: Regression of SLP (shadings) and vertically integrated moisture transport (vectors) on −SIE09hp for each month over the years of 1979–2013. The data are from observation as well as the ERA-Interim reanalysis. Maps were generated using the "matplotlib basemap toolkit" Python package (`https://matplotlib.org/basemap/`), version 1.1.0.

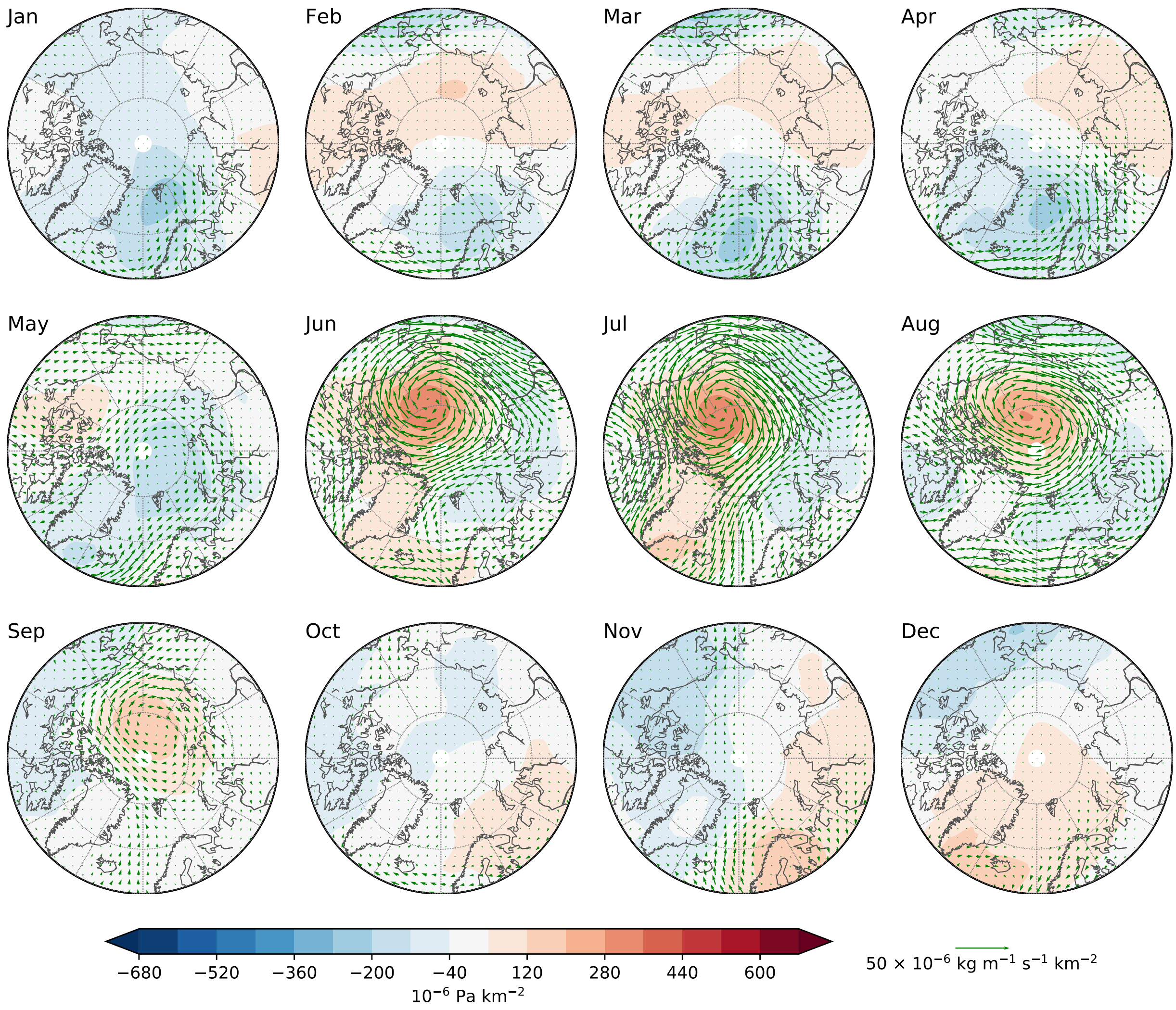

Figure S6: Same as Figure S5 except the data are ensemble-mean regressions from CESM-LE. Maps were generated using the "matplotlib basemap toolkit" Python package (`https://matplotlib.org/basemap/`), version 1.1.0. Maps were generated using the "matplotlib basemap toolkit" Python package (`https://matplotlib.org/basemap/`), version 1.1.0.

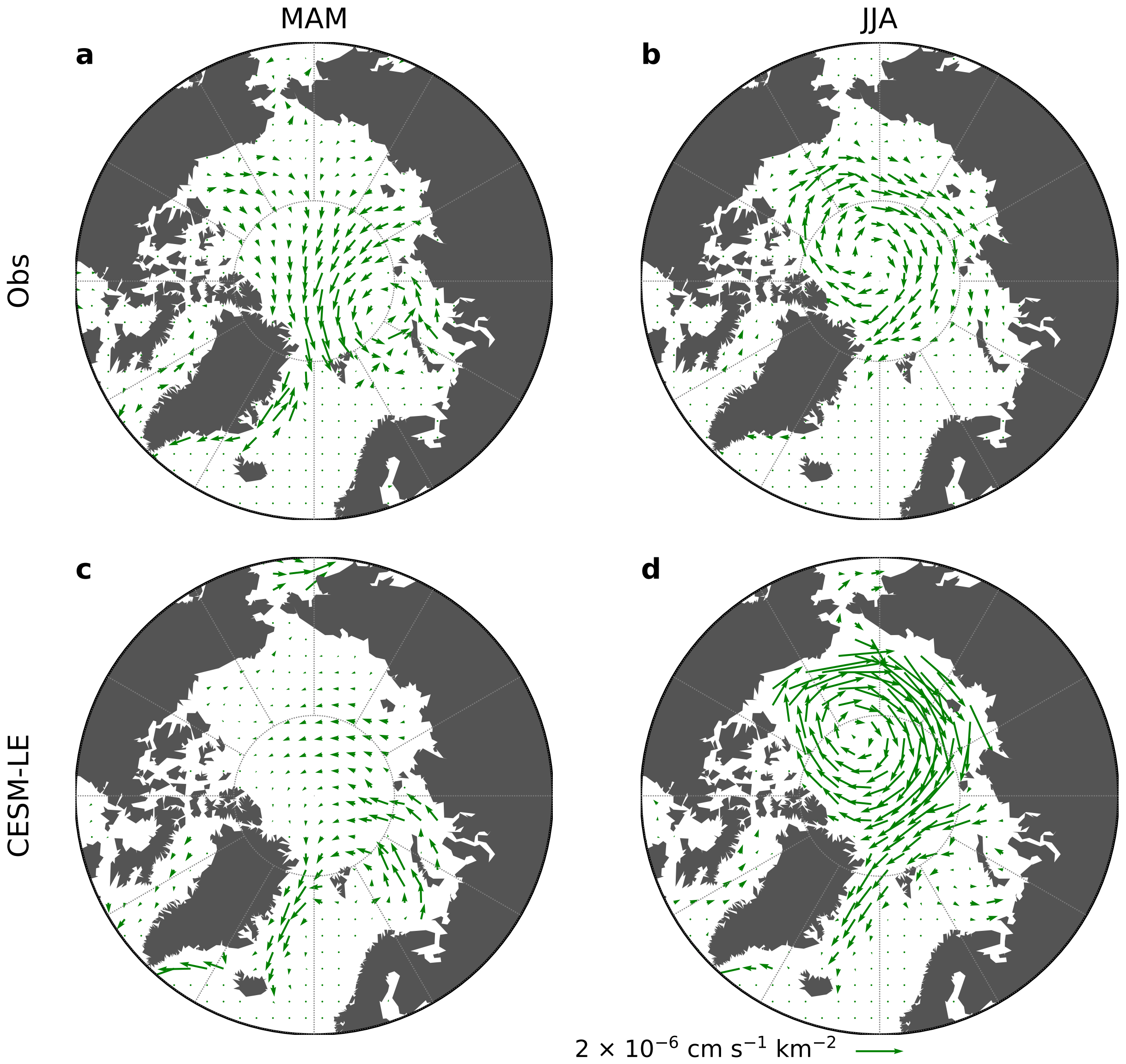

Figure S7: Regression of sea ice motion on −SIE09hp over the years 1979–2013 for season MAM (a and c) and JJA (b and d). (a) and (b) show the results from observations while (c) and (d) are ensemble-mean regressions from CESM-LE. Maps were generated using the "matplotlib basemap toolkit" Python package (`https://matplotlib.org/basemap/`), version 1.1.0.

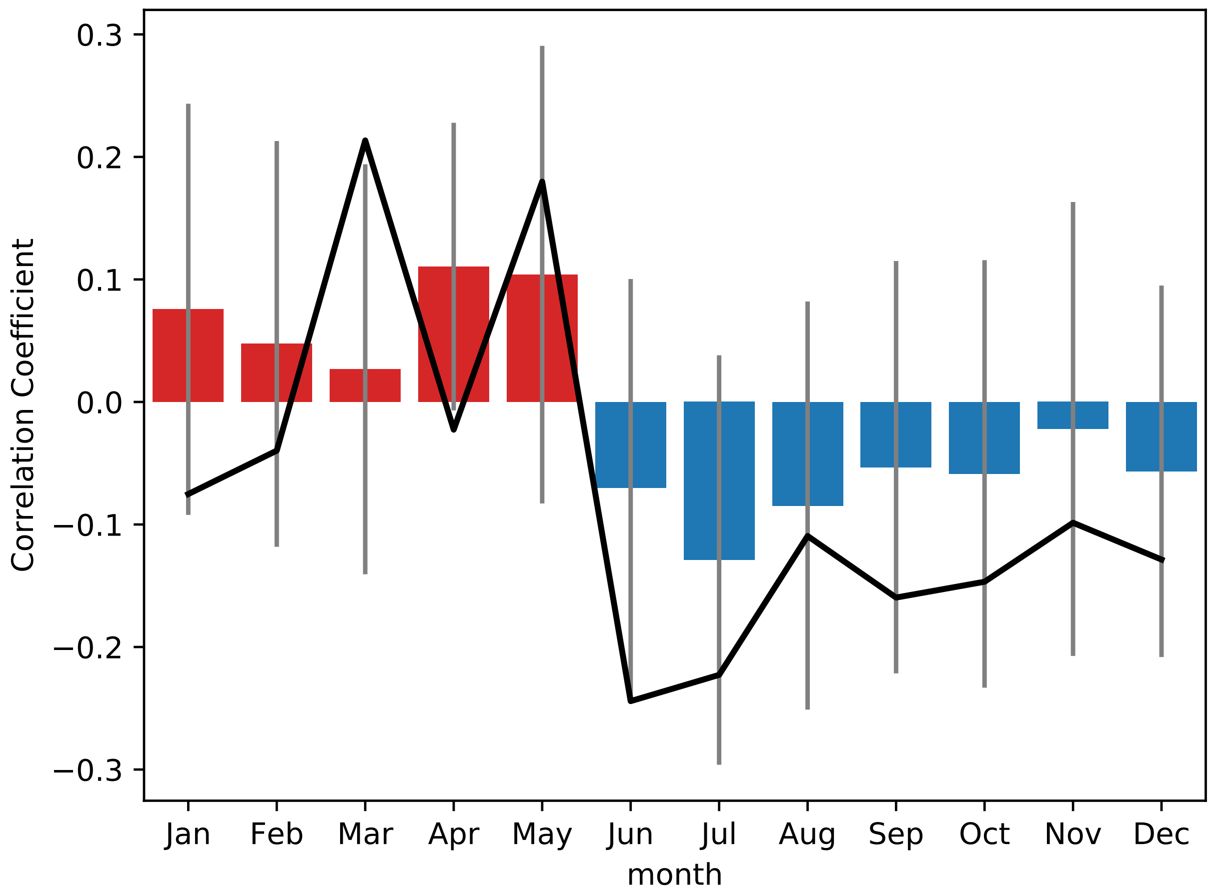

Figure S8: Correlation between the zonal-mean vertically integrated meridional moisture transport at 70N and −SIE09hp in observation (black line) and CESM-LE (bars and gray lines) for each month. The bars represent ensemble mean, while the vertical gray lines show the spread of one standard deviation from the ensemble members. Hatches denote ensemble-mean values significantly different from zero at 0.05 level by the two-sided Student's t-test.

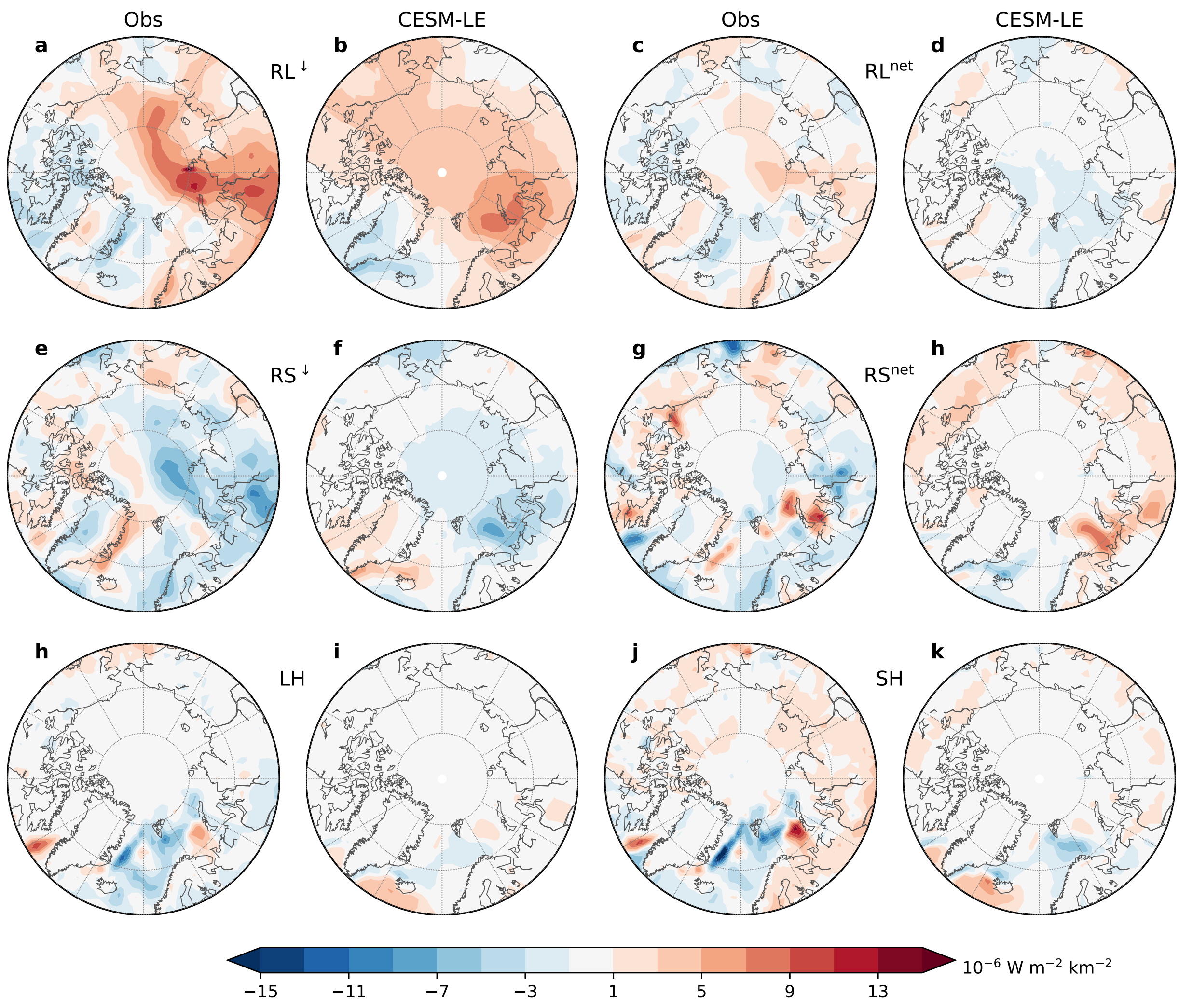

Figure S9: Regression of MAM surface heat fluxes on $-$SIE09hp over the years of 1979–2013 for observation and ensemble-mean regressions from CESM-LE. The heat fluxes include: downward longwave radiation ($RL^{\downarrow}$, a and b), net longwave radiation (RL^{net}, c and d), downward solar radiation ($RS^{\downarrow}$, e and f), net solar radiation (RS^{net}, g and f), latent heat flux (LH, h and i) and sensible heat flux (SH, j and k). The first and third columns show results from observations while the other two columns are for CESM-LE. Maps were generated using the “matplotlib basemap toolkit” Python package (`https://matplotlib.org/basemap/`), version 1.1.0.

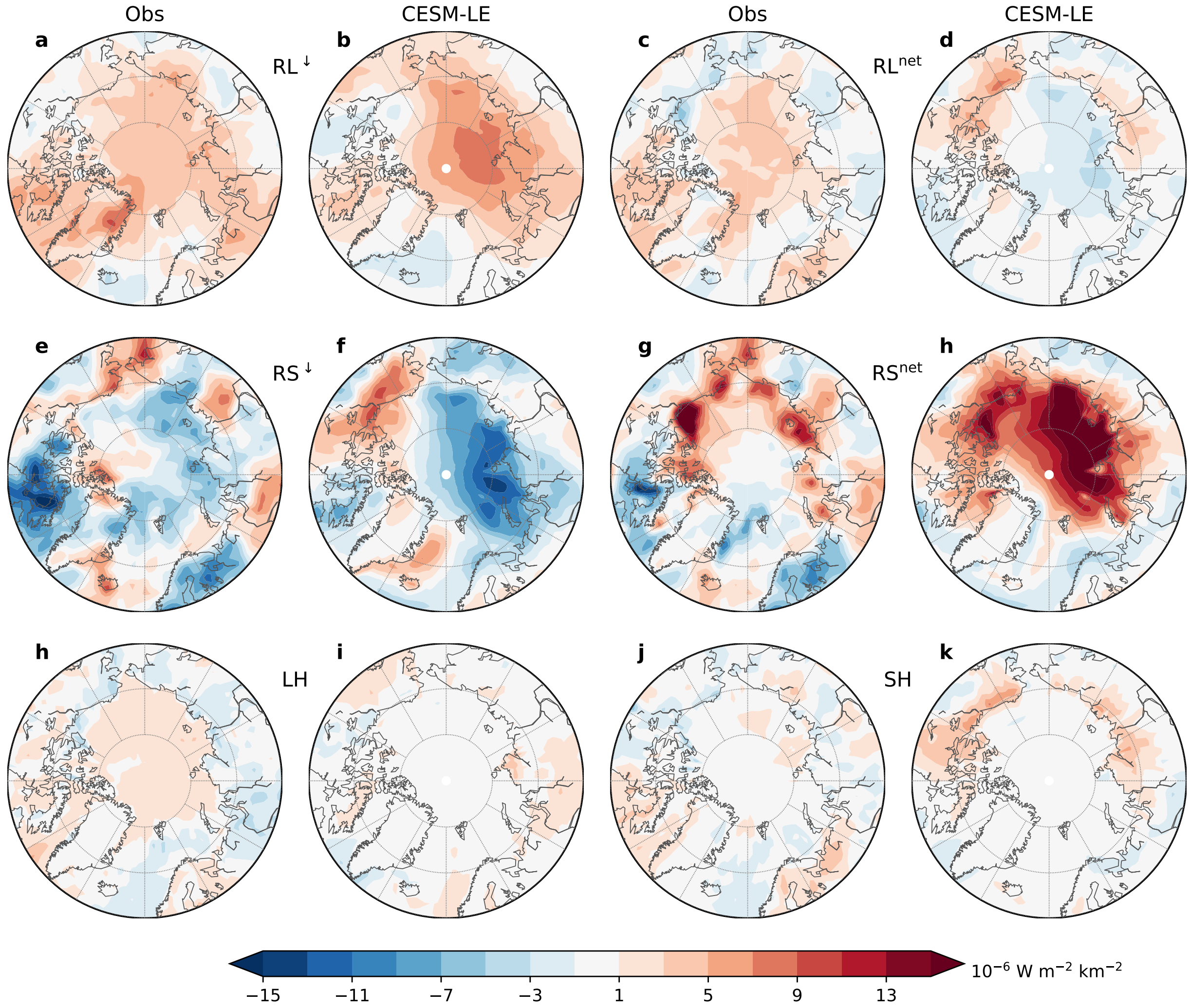

Figure S10: Same as Figure S9 except for the season of JJA. Maps were generated using the "matplotlib basemap toolkit" Python package (`https://matplotlib.org/basemap/`), version 1.1.0.
